# Optimization of the arginase activity assay micromethod for macrophages and sera

**DOI:** 10.1186/s13104-023-06462-4

**Published:** 2023-08-29

**Authors:** Romaric Nzoumbou-Boko, Cyrille Oliver Ozzin-Kholy Zolipou, Brice Martial Yambiyo, Silla Semballa, Mireille Cornelia Ingrid Denissio Morissi Nalingbo, Sylvie Daulouède, Philippe Vincendeau

**Affiliations:** 1 Laboratoire de Parasitologie, Institut Pasteur de Bangui, BP 923, Bangui, Central African Republic; 2 Laboratoire de Parasitologie, UMR 177 IRD/CIRAD “INTERTRYP,” Université Bordeaux, Bordeaux, F-33000 France; 3 Service d’Epidémiologie, Institut Pasteur de Bangui, BP 923, Bangui, Central African Republic; 4 Laboratoire des Sciences Biologiques et Agronomiques pour le Développement (LASBAD), Université de Bangui, République Centrafricaine, Bangui, Central African Republic

**Keywords:** Arginase activity, Micromethod optimization, Trypanosomiasis

## Abstract

**Objective:**

We optimized the spectrophotometric micromethod for the determination of arginase activity based on the Corraliza et al. modification of Schimke’s method. Arginase activity in sera from patients suffering from human African trypanosomiasis, in macrophage lysates from trypanosome-infected mice, and in purified bovine liver arginase was compared using the conventional and optimized micromethods.

**Results:**

The sensitivity of both micromethods was comparable. However, our optimized method has the following advantages: it uses small sample volumes (6 µl per assay vs. 50 µl) and reagent volumes (200 µl vs. 400 µl), it can be carried out in a single microplate well, thereby minimizing handling, and it requires fewer materials and utilizes readily available equipment. Our optimized method proved to be applicable and well suited for small-volume samples and resource-poor laboratories.

## Introduction

Arginase activity is generally associated with liver function, involved in the detoxification of ammonia in the urea cycle and the production of ornithine needed for the synthesis of proline and polyamines, essential for cell growth, DNA synthesis, and collagen synthesis [1]. In addition, arginase competes with nitric oxide synthase (NOS) for the common substrate L-arginine, and regulatory interactions between arginase and NOS are involved in a variety of biological functions. Furthermore, arginase has been linked with various diseases, including those affecting the central nervous system, kidneys, the cardiovascular system, as well as immune dysfunction and cancer [1]. The essential role of arginase and inducible L-arginine metabolism in infectious diseases has been demonstrated in experimental trypanosomiasis and leishmaniasis and has since emerged as playing an important role in infections such as human African trypanosomiasis (HAT), AIDS, malaria, human leishmaniasis and COVID-19 [2–5]. Arginase induction is the hallmark of the type 2 immune response and alternative activation of macrophages and is also a marker of myeloid-derived suppressor cell activity.

The multifunctional and multifaceted enzyme arginase and its implication in various physiological processes and pathophysiological conditions have led to numerous studies on gene expression, enzyme characterization, and enzyme activity. Several analytical methods have been developed for the determination of arginase activity, including Schimke’s method improved by Corraliza et al. [6]. Since 1969, the determination of arginase activity in blood serum has evolved to adapt to other types of specimens or samples, using other techniques or materials. For example, fluorometric micromethods have been developed for dried blood spots on filter paper and ELISA arginase assays are used to detect the arginase protein in sera [7]. A radioactive assay using ^14^ C L-arginine is another option [8].

The assessment of arginase activity with the Corraliza (modified Schimke’s method) micromethod is one of the most commonly used methods. This micromethod was initially employed to evaluate arginase activity in macrophages from infected mice and then in sera from HAT patients, as a biomarker of treatment efficacy [2, 9]. The conventional Corraliza micromethod requires 50 µl of sample, several transfers between different types of tubes, and various pieces of relatively large equipment such as a water bath, an incubator, an oven, and spectrophotometer, making this procedure relatively labor-intensive.

In this paper, we describe improvements on this micromethod to allow its application in laboratories with limited means or in field conditions, using purified enzyme, blood and cell extracts.

## Main text

### Methods

Bovine liver arginase (L-arginine amidinohydrolase, EC 3.5.3.1), L-arginine, Triton X-100, Tris, HEPES, MnCl_2_, protease inhibitor mixture, α-isonitrosopropiophenone, 96% sulfuric acid, orthophosphoric acid, and ethanol were all purchased from Sigma-Aldrich Chimie (Saint-Quentin-Fallavier, France).

Animals were housed according to institutional guidelines. Experiments and care of mice complied with guidelines of the European Convention for the Protection of Vertebrate Animals used for Experimental and other Scientific Purposes (CETS n° 123). Experiments were approved by the Department for the Protection of Animals and Plants of the Préfecture de la Gironde. This study was approved by the Bordeaux Ethics Committee 50 (number CE50-RE: B 33,063,324) and was carried out in accordance with the 1964 Declaration of Helsinki and its later amendments.

Female Swiss mice (OF1) eight to ten weeks old, 18–25 g, (Charles River, L’Arbresle, France) were kept fifteen days before the experiment was started in our animal housing facility in ventilated boxes kept in a protected temperature and humidity-controlled room, with a 12 h on/off light cycle. They were housed in standard acrylic transparent cages (36.5 × 20.7 × 14 cm), seven mice to a cage, containing wooden bedding, a plastic nest box, nesting material (cotton nestlets) and a cardboard roll for environmental enrichment (SAFE CAC, Augy, France). Food (Scientific diet for mice, SAFE AO4 SP) and water were provided ad libitum. Efforts were made to minimize the suffering of animals used.

*Trypanosoma musculi* (*Partinico II strain*) was stored in liquid nitrogen or maintained in vivo by intraperitoneal injection into naive mice [10]. *T. musculi* is a natural murine parasite, leading to a limited number of blood parasites, well tolerated in mice. *T. musculi* infection has been considered as an appropriate model of the host-parasite relationship [11, 12].

Two groups of seven mice / cage were then randomly distributed:

- One group (control group) received physiological saline (0.5 mL) by intra-peritoneal injection;
- One group received (5 × 10^4^ parasites/ physiological saline (0.5 mL) per mouse) by intra-peritoneal injection.

Thus, the total number of animals used was 14 and there were no exclusions according to recommendations of local ethic committee the number of animals was kept to the minimum. Mice were killed by manual cervical dislocation and peritoneal macrophages from mice infected with *T. musculi* (n = 7) and uninfected mice (control macrophages) (n = 7) were collected, at days 13 postinfection and arginase activity was measured ex vivo macrophages from uninfected mice or infected animals. Briefly, 100,000 macrophages/well were plated on a 96-well microplate (Greiner), lysed in a cocktail solution containing a Triton X-100 and protease inhibitor mixture, and stored at − 80 °C until use as previously described [9].

Sera from HAT patients (n = 10) and healthy controls (n = 10) were sampled during a mass screening for sleeping sickness in the endemic area of Boko-Songho (Bouenza Region, Republic of Congo) and were stored at − 80 °C until use, the scientific protocol was approved and authorized by the Congo Ministry of Health and informed consent was obtained from patients [13].

L-arginine amidinohydrolase was diluted in deionized water and a range of reference arginase standards was made (0, 1, 5, 10, 20 and 40 U/ml). Arginase assays on reference enzyme dilutions, macrophage lysates, and sera were carried out in triplicate using the two protocols: the conventional Corraliza micromethod and our optimized micromethod, according to experimental conditions described in Table 1. The Greiner-type 96-well microplate (Fisher Scientific, Illkirch, France) with excellent thermal and chemical stability, was employed in this study. Absorbance was measured at 550 nm directly in the plates using a spectrophotometer (SPECTROstar Nano, BMG Labtech, Ortenberg, Germany). A thermocycler GeneAmp, PCR system 9700 (Applied Biosystems, Thermo Fisher Scientific, Paisley, United Kingdom) was used for the various water bath, incubation and heating steps in the optimized micromethod.

**Table 1:** Comparison of the different steps of the two techniques

	Micromethod	Optimized micromethod
**Equipment**	Waterbath, Oven, Incubator and Spectrophotometer	Thermocycler and Spectrophotometer
**Activation**	Sample volume: **50 µL** Buffer : **Tris** **Waterbath** : 56 °C for 15 min Eppendorf tube	Sample volume: **6 µl** Buffer: **HEPES** **Thermocycler** : 56 °C for 15 min Microplate well
**Reaction**	Substrate: Arginine in **PBS** **Proofer** at 37 °C for 1 h 30 Addition of **400 µL** acidic mix Eppendorf tube	Substrate: Arginine in **deionized water Thermocycler** à 37 °C for 1 h 30 Addition of **200 µL** acidic mix Same Microplate well
**Revelation**	Addition of **25 µL** ISPF **Oven** at **100 °C** for 45 min Glass tube	Addition of **15 µL** ISPF **Thermocycler** at **96 °C** for 45 min Same Microplate same well
**Reading**	Spectrophotometer 550 nm Microplate well	Spectrophotometer 550 nm Same Microplate well

For the enzymatic assays of arginase, the statistical analyses were carried out using STATA software (version 14; Stata Corp, College Station, Texas, USA) and were performed using Welch’s *t*-test, an adaptation of Student’s *t*-test for comparison of small sample means with unequal variances; *P-*values of < 0.05 were considered significant. Intergroup comparisons for activated macrophages vs. control macrophages and HAT sera vs. control sera were tested using the nonparametric Mann-Whitney U test; *P‐*values of < 0.05 were considered significant. A Kruskal-Wallis test was used to compare the two assay methods.

## Results

**Fig. 1:**
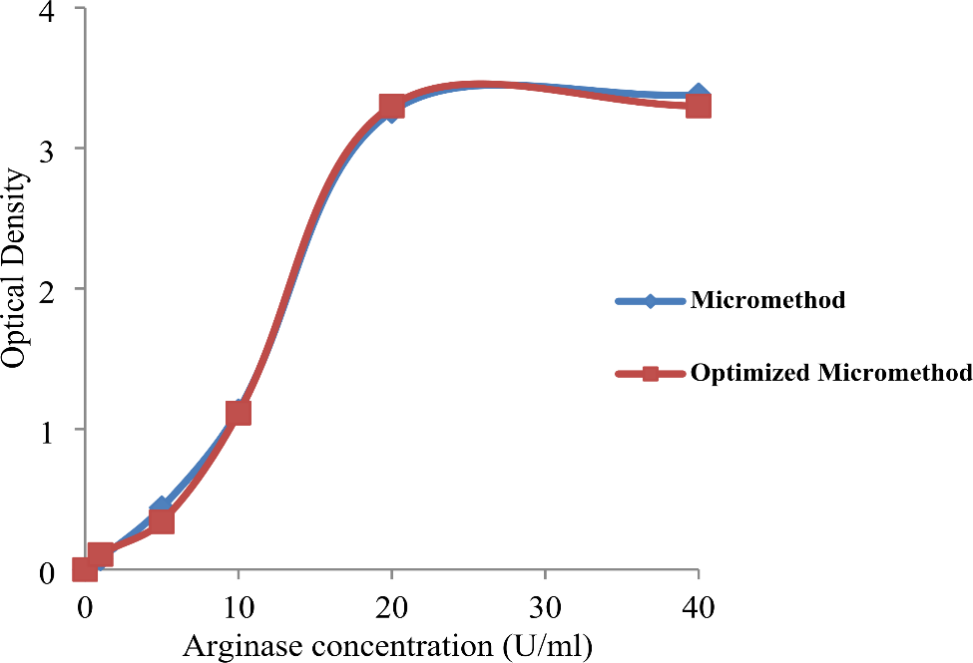
Enzymatic Assay of Arginase with two procedures

Results from the two methods were very similar with a *P-*value of 0.98, indicating no statistically significant difference (Fig. 1).

**Fig. 2:**
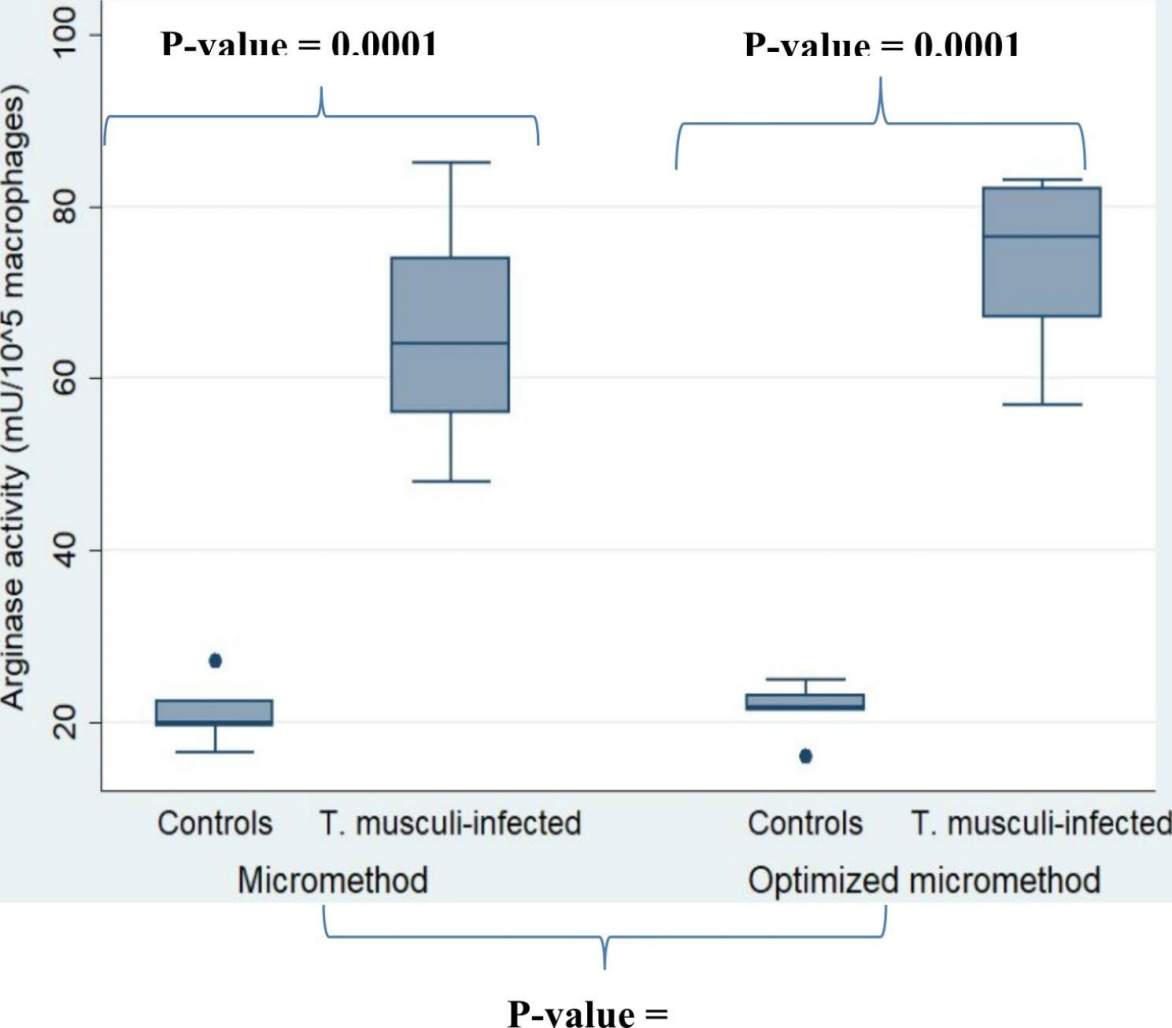
Use of the non-parametric Mann-Whitney test for intergroup comparisons between activated macrophages vs. control macrophages and HAT sera versus control sera. For the comparison of the two methods, the Kruskal-Wallis test is used for determination of arginase activity in macrophage mouse controls (n = 7) and macrophage mouse *T. musculi* infected (n = 7)

The arginase activity was higher in macrophages from infected mice than in control macrophages in both assay methods (*P-*value = 0.0001) (Fig. 2), and there was no statistical difference between the two methods (*P-*value = 0.34).

**Fig. 3:**
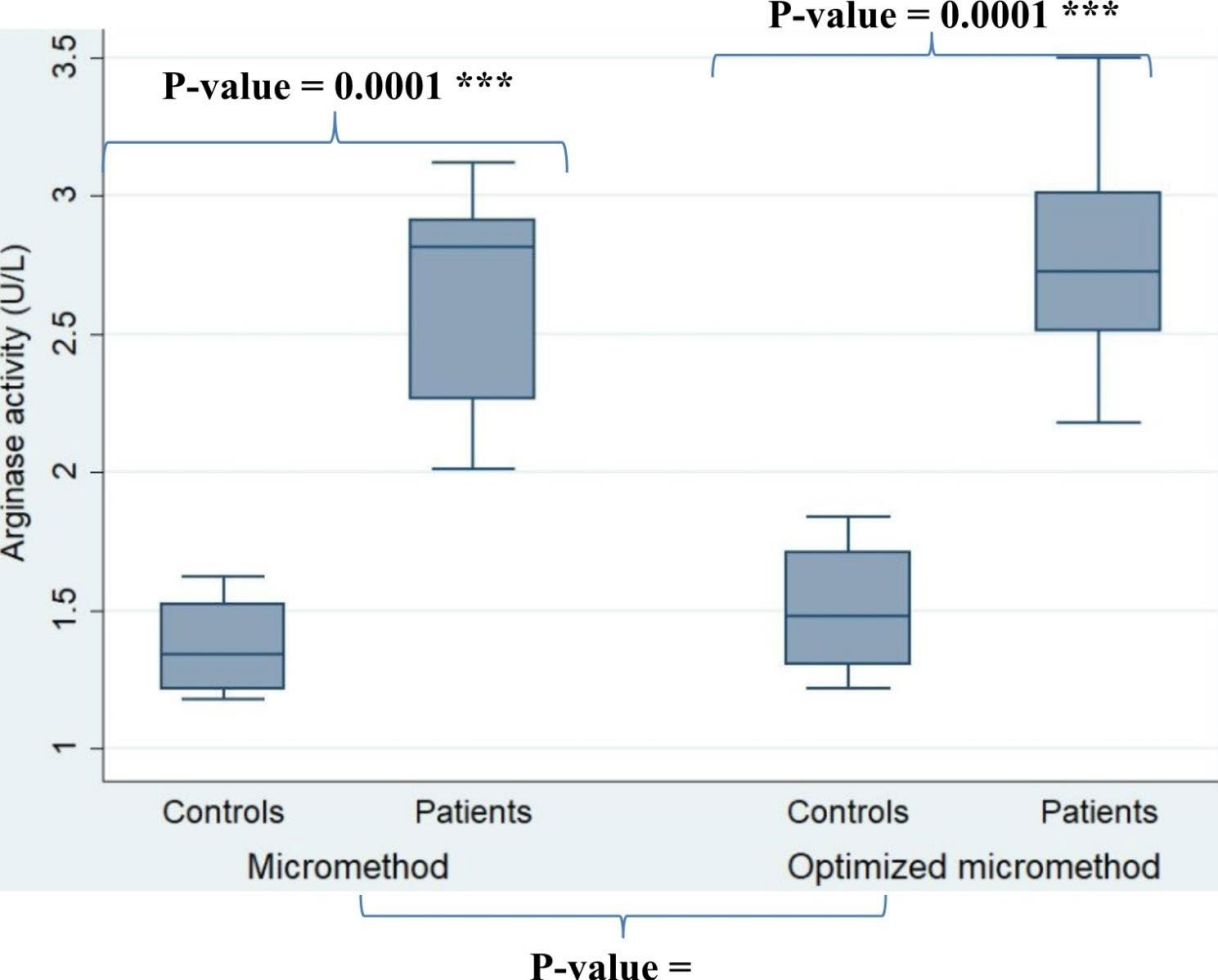
Use of the non-parametric Mann-Whitney test for intergroup comparisons between activated macrophages vs. control macrophages and HAT sera versus control sera. For the comparison of the two methods, the Kruskal-Wallis test is used for determination of arginase activity in sera from controls (n = 10) and patients with human African trypanosomiasis (n = 10) from the Bouenza focus in the Republic of Congo

Arginase activity was significantly higher in sera from HAT patients than in control sera with (*P-*value = 0.0001) (Fig. 3), and there was no statistical difference between the methods (*P-*value = 0.99).

## Discussion

Several analytical methods have been developed for the determination of arginase activity in biological samples. Here, we optimized the Corraliza micromethod by reducing sample volume and minimizing handling and pipetting. Both methods gave similar patterns of enzyme activity in the reference purified bovine liver arginase. Similarly, both methods equally detected increased induced arginase activity in macrophages from infected mice and increased levels of arginase activity in HAT patient sera, compared with their respective controls.

Reducing the required sample volume conserves precious biological samples and allows for longitudinal studies over time on the same individual laboratory animal, thereby reducing the number of experimental animals needed. In our experiments, a thermocycler was used in place of a water bath, an oven, and an incubator. Thermocyclers are now present in the majority of medical analysis laboratories, even in organizations with limited means. Along with reducing reaction and reagent volumes, all the steps take place in the same 96-well microplate, whereas in the conventional micromethod, some steps require microtubes and glass test tubes, thus requiring transfers. Moreover, all samples can be stored and analyzed in the same microplate.

Although powerful techniques are available for the study of the arginase pathway and its involvement in immunology and physiology, the determination of arginase activity is important. Other techniques, such as real-time PCR, quantify only the gene count, and still others, such as ELISA, only assess the concentration of the arginase protein [6, 14].

Improvement in arginase measurements in various pathophysiological conditions can contribute to the investigation of the complex arginine metabolism in different tissues, biological fluids and biopsies.

The determination of arginase activity is of importance in clinical protocols, therapeutic monitoring, and assessment of disease severity. High arginase activity is a marker of severe visceral leishmaniasis, particularly in co-infection with HIV infection [15]. Recently, severe COVID-19 has been associated with elevated levels of arginase, which thus may represent a promising marker in the pathogenesis of this disease [5, 16].

## Conclusion

Here, an optimized procedure of the conventional micromethod was developed to determine arginase activity with reduced sample and reagent volumes, limited handlings, few materials and limited equipment. The assayed arginase activity is statistically comparable between the optimized and conventional micromethods, thereby offering equal sensitivity. This micro-assay method can be applied to various biological samples to further investigate arginine metabolism in various pathophysiological conditions.

## Limitations

This study is limited by the small sample size. Other limitations of the study include the sera that were collected and stored at -80 °C in 2004 and also that the thermocyclers previously used in research laboratories for amplifying DNA is now most widely used in routine for diagnostic tests for detecting pathogens.

## Data Availability

The datasets used and/or analyzed during the current study available from the corresponding author on reasonable request.
